# Transient Systolic Anterior Motion of the Anterior Mitral Valve Leaflet in a Critical Care Patient with a Structurally Normal Heart

**DOI:** 10.7759/cureus.3963

**Published:** 2019-01-25

**Authors:** Hassan Abbas, Sriviji Senthil Kumaran, Muhammad A Zain, Asrar Ahmad, Zain Ali

**Affiliations:** 1 Internal Medicine, Abington Memorial Hospital, Abington, USA; 2 Internal Medicine, Sri Muthukumaran Medical College, Chennai, IND; 3 Internal Medicine, Sheikh Zayed Medical College and Hospital, Rahim Yar Khan, PAK

**Keywords:** systolic anterior motion, transient systolic anterior motion, sam in structurally normal heart, sam, catecholamine exposure and hypotension with systolic murmur, hypotension with murmur in icu patient, hypotension in critical care patient

## Abstract

Systolic anterior motion (SAM) is defined as the displacement of the anterior mitral leaflet towards the left ventricular outflow tract, which results in left ventricular outflow tract obstruction (LVOTO). The SAM of the anterior mitral leaflet is a well-established phenomenon in hypertrophic obstructive cardiomyopathy (HOCM), but its occurrence in a structurally healthy heart is uncommon. We present a critical care patient with presumed septic shock whose blood pressure was previously controlled by fluid resuscitation and vasopressors. He developed a new cardiac murmur along with hypotension despite being on vasopressors. The echocardiographic assessment revealed no structural heart disease or valvular vegetations but a hyperdynamic left ventricle with significant SAM of the anterior mitral leaflet, resulting in mitral regurgitation (MR). The murmur and hypovolemia resolved after aggressive fluid resuscitation and by decreasing the vasopressor dose.

## Introduction

Left ventricular outflow tract obstruction (LVOTO) secondary to systolic anterior motion (SAM) of the anterior mitral valve leaflet was first described in patients with hypertrophic cardiomyopathy (HCM). However, cases of SAM of anterior mitral valve leaflet (AMVL) without underlying HCM have been reported in critical care patients secondary to undesirable risk factors, such as hypovolemia or excessive catecholamine exposure [1]. Herein, we present a case of septicemia previously managed successfully with vasopressors and fluid resuscitation, who went on to develop hypotension and new onset cardiac murmur secondary to inotropes exposure. Echocardiography revealed LVOTO secondary to SAM of AMVL. The murmur and hypotension were treated with fluid resuscitation and by decreasing the dose of vasopressors. Our case highlights the rare LVOTO related to catecholamine exposure in intensive care unit (ICU) patients with underlying septic shock and hypovolemia. We also report the successful management of lethal hypovolemia related to SAM with aggressive volume resuscitation and weaning off the vasopressors.

## Case presentation

A 63-year-old male with a past medical history of Crohn’s disease, alcoholism, and stage III chronic kidney disease was found hallucinating by his wife. Emergency medical services (EMS) was called, and the patient was found immobilized with his left leg caught between his bed and the adjacent wall. En route, the patient developed pulseless wide complex tachycardia requiring defibrillation. Return of spontaneous circulation was achieved after one epinephrine injection. He was intubated shortly after that. On arrival to the emergency room (ER), the patient was found to be febrile (103*F), hypotensive with a blood pressure of 90/60 mmHg, and oxygenating well on minimal ventilatory settings. He was sedated due to agitation. The physical exam showed a deep open ulcer on the lower left leg (LLL) and a gangrenous-appearing ipsilateral foot. All other systems were otherwise healthy. No murmurs were heard on auscultation.

Investigation

He had severe derangement of his lab workup, as follows: Creatinine (Cr) of 8.07 (0.6 to 1.2 milligrams per deciliter normal), blood urea nitrogen (BUN) of 90 (7 to 20 mg/dL normal), sodium of 171 milliequivalents per liter (mEq/L) (135 to 145 mEq/L normal), potassium of 4.9 mEq/L (3.5-5.0 mEq/L normal), and serum bicarbonate of 11 mEq/L with an anion gap of 40 and an osmolar gap of 27. His complete blood count (CBC) showed white blood count (WBC) 10,900 per cubic milliliter with 69% neutrophils (4000 and 11,000 normal), hemoglobin (Hb) 16.8 g/dL, hematocrit 54.7%, and platelets 77,000 per microliter (150,000 to 450,000 per microliter normal). His troponin was elevated at 0.78, creatinine kinase (CK) was 5029 unit per liter (22 to 198 U/L), aspartate transaminase was (AST) 298 U/L (10 to 40 U/L normal), and alanine transaminase (ALT) was 94 U/L (7 to 56 U/L). A serum and urine drug screen was obtained and was negative, including an undetectable serum alcohol level. Electrocardiogram (EKG) showed sinus tachycardia. An arterial blood gas test (ABG) revealed a pH of 7.17 with appropriate respiratory compensation to the metabolic acidosis.

Treatment

The patient was started on empiric antibiotics due to the anticipation of septic shock. After initial resuscitation with five liters of normal saline, his blood pressure improved. Acute kidney injury related to toxic alcohol ingestion was suspected, given his long-standing history of alcoholism. Nephrology consultation recommended temporary hemodialysis overnight, which resulted in a rapid improvement in his acid-base and electrolyte disturbances. Methanol and ethylene glycol levels, sent upon admission, returned undetectable on Day 2. It was also thought that rhabdomyolysis could have occurred secondary to immobility in a sustained posture, which might have lead to myoglobinuria-related acute tubular necrosis. The elevated CK supported this diagnosis. The patient was evaluated by surgery for the necrotic foot and the determination of dry gangrene was made. A plan for eventual amputation was made once he became stable. He continued to make progress and did not require dialysis after the first night. Urine output improved after the early 18 hours with anuria. On Day 5, his basal metabolic profile showed BUN 8 and Cr 0.89. However, the patient's mental status continued to wax and wane, and he repeatedly failed attempts to wean off the ventilator. Blood cultures returned negative.

On Day 5, the patient's hypotension warranted vasopressors. A repeat blood culture was collected and the antibiotics coverage broadened, suspecting septic shock, with the gangrenous foot being the likely source - an assumption with which the surgeons did not concur. His repeat CBC showed WBC 5700 (51% neutrophils), Hb 9.4 g/dL, hematocrit 27.5%, and platelets 239,000 per microliter. His blood pressure maintained well on vasopressors throughout Day 5. The next morning, the patient became hypotensive again despite being on vasopressors. A holosystolic murmur was heard on auscultation. This murmur was a new finding, which had not been present on admission or until that day. It was a grade III/VI holosystolic murmur, best heard at the apex, with radiation to the axilla. Valsalva and handgrip tests were not performed since the patient was sedated and intubated. An electrocardiogram (EKG) was ordered, which did not show any changes indicative of ischemia. Troponin levels were repeated and were not detectable. A transthoracic echocardiogram (TTE) was ordered after a cardiology consultation to rule out infective endocarditis. The echocardiogram revealed a hyperdynamic left ventricle and SAM of the anterior mitral leaflet, resulting in LVOTO (Video 1) and severe mitral regurgitation evident on Doppler imaging (Video 2).

**Video 1:** Hyperdynamic LV and SAM of the Anterior Mitral Valve Leaflet LV: left ventricle, SAM: systolic anterior motion

**Video 2:** Doppler Imaging Showing Severe MR MR: mitral regurgitation

In contrast, an echocardiogram done on Day 2 of admission showed normal left ventricular systolic function and no valvular disease. It was suspected that he developed the post-acute kidney injury (AKI) diuresis phase during the fourth to sixth days of admission, which resulted in hypovolemia. On top of that, he developed dynamic LVOTO due to catecholamines exposure. As per the diagnosis of SAM of the anterior mitral leaflet, more aggressive fluid resuscitation, along with decreasing the vasopressor dose as tolerated, was advised. The repeat blood cultures continued to be negative.

Outcome and follow-up

After the institution of interventions as mentioned above, the patient's blood pressure improved, vasopressors were weaned, and the systolic murmur disappeared. The patient was also weaned off the ventilator later on. The hyperdynamic left ventricle was thought to be a result of post-AKI diuresis, rendering him hypovolemic, with the administration of vasopressors making matters worse. A repeat echocardiogram closer to discharge revealed normal left ventricular systolic function without a hyperdynamic left ventricle (Video 3) and trace mitral regurgitation was seen on Doppler imaging, which may be physiologic (Video 4). The septum was normal in thickness, and no regional wall motion abnormality was detected, which confirmed the transient nature of the process during periods of hyperdynamic circulation.

**Video 3:** Normal LV Systolic Function Without Hyperdynamic LV LV: left ventricle

**Video 4:** Doppler Showing Normal LV Systolic Function with Trace MR LV: left ventricle, MR: mitral regurgitation

## Discussion

Systolic anterior motion (SAM) of the mitral valve is described as a condition where the mitral valve leaflet is displaced anteriorly, during systole, to the left ventricular outflow tract (LVOT). Although not a requirement for the diagnosis, it has always been considered a classic feature of hypertrophic cardiomyopathy (HCM).

SAM of the mitral valve can present itself in very different ways. The presentation can range from a morphological echocardiographic abnormality to a history of a new cough and dyspnea, representing pulmonary congestion, to a severe hemodynamic collapse due to an LVOT obstruction (LVOTO) and mitral regurgitation [2]. The prevalence of SAM of the mitral valve in patients without pre-existing cardiac disease is not well-studied but is thought to be <1% [1].

On reviewing the literature, SAM of the mitral valve in patients without HCM has now been found in a variety of other clinical scenarios. Mitral valve repair [3] and aortic valve replacement [4] are two critical surgical procedures that are associated with the development of SAM. Comorbid conditions, such as hypertension [5] and diabetes [6], can also give rise to SAM due to increased catecholamine sensitivity. Cases have also been reported in those who have had a recent myocardial infarction [7], healthy adults undergoing dobutamine stress test [8-9], or when under general anesthesia [10]. There have been four previous reports of the use of vasopressors in septic shock, resulting in SAM and LVOTO and worsening hemodynamic stability [11-14]. One fatal case has been reported in which vasopressor-induced SAM of the mitral valve resulted in lethal hypovolemia [15]. A study conducted by Chockalingam et al. [16] emphasized the underdiagnosis of dynamic left ventricular outflow tract obstructions in the critical care setting. Therefore, a very high degree of clinical suspicion is essential to diagnose dynamic outflow obstructions in a patient. Our patient developed SAM with the use of vasopressor in a volume-repleted state.

The exact mechanism by which SAM develops is still incompletely understood. Some morphological features of the mitral valve and the left ventricle cause a decrease in the coaptation - septum distance, thereby increasing the likelihood of SAM [17]. Why this phenomenon occurs in certain individuals and not in others is a matter that may need further investigation. Sabzwari et al. [18] attributed this phenomenon to an elongated anterior mitral valve leaflet in their patient. However, this abnormality was not seen in our case. On the other hand, an increase in flow velocity across the mitral valve is of more importance in our patient. Previous studies [8,11] have shown that in an underfilled heart with positive inotropic effect, the flow across the valve is of increased velocity, which creates a drop in pressure, pulling the mitral valve leaflets towards the septum, that is, the venturi effect. Another possibility is that in some patients, the mitral valve leaflets are prepositioned in the path of flow, which causes a drag effect, further positioning them anteriorly and superiorly towards the septum. Hypovolemia and excess inotropes were found to be the trigger in our patient, as both of these increase the flow velocity [17].

Most of the vasopressors used in the critical care setting do have ionotropic effects. They can also have direct toxic effects on myocyte [8,19-20]. Using these drugs before addressing intravascular volume deficits can increase contractility in a volume-depleted and small left ventricle, stimulating the onset of LVOT obstruction [16].

The typical clinical manifestations are cough, dyspnea, and angina [15]. Vital signs show tachycardia and hypotension. The ECG could show ST-T changes suggestive of coronary disease [15]. The murmur heard is the only consistent finding [16]. It is a systolic murmur heard best in the left third intercostal space and augments with Valsalva.

Diagnosis is established by echocardiography that will show the SAM of the anterior mitral leaflet. Doppler can quantify the LVOT obstruction. Since dynamic LVOT obstruction is associated with increased mortality [15], early identification and prompt treatment are essential in critical care patient. The possible differentials are infective endocarditis (especially when sepsis is strongly suspected), myocardial infarction and its mechanical complications, like ruptured interventricular septum, papillary muscle rupture, or the rarer acute MR secondary to the spontaneous rupture of the chordae tendineae.

The patients are treated with fluid resuscitation and by withdrawing the offending inotropic agent. Theoretically, interventions such as beta blockers, nondihydropyridine calcium channel blockers, or switching to a vasopressor agent with selective alpha agonist properties (such as phenylephrine) can be used, which will not only lower cardiac inotropy but provide extended LV filling time, thereby relieving LVOTO. However, these drugs should be used with caution in these hypovolemic individuals, as they can worsen cardiogenic shock. Therefore, a more significant study is yet to testify these treatment modalities in a bigger picture [16].

## Conclusions

It is pertinent to revisit the pathophysiology and presentation of systolic anterior motion (SAM) of the mitral valve leaflet in patients other than those with HCM. Transient SAM due to vasopressor stimulation in a volume-deficient individual can cause dynamic left ventricular outflow obstruction. It is a frequently missed diagnosis in critical care patients. This condition should be suspected in a critical care patient who presents with hypotension and a systolic murmur. Identification with echocardiography and the prompt correction of volume status along with the weaning off catecholamines is the preferred treatment, with better outcomes.
